# Triple Bypass for Chronic Pancreatitis With Biliary and Duodenal Stenosis: A Report of Two Cases

**DOI:** 10.7759/cureus.82738

**Published:** 2025-04-21

**Authors:** Aya Noguchi, Masaharu Ishida, Daisuke Douchi, Masamichi Mizuma, Michiaki Unno

**Affiliations:** 1 Department of Surgery, Tohoku University Graduate School of Medicine, Sendai, JPN

**Keywords:** bile duct stenosis, chronic pancreatitis, duodenal stenosis, lateral pancreaticojejunostomy, longitudinal pancreaticojejunostomy

## Abstract

Pancreaticoduodenectomy is typically performed for patients with biliary and duodenal stenosis due to chronic pancreatitis. However, the procedure can be quite challenging in cases with severe inflammation and adhesions and may be too invasive for patients in poor general condition. We report two cases of triple bypass, a procedure that combines pancreatic duct drainage with biliary and gastric bypass, as an alternative to pancreaticoduodenectomy. Case 1 involves a 51-year-old man who underwent endoscopic pancreatic and biliary stenting for pancreatic and biliary stenosis caused by alcoholic chronic pancreatitis. Although coexistent duodenal stenosis was observed during upper gastrointestinal endoscopy, he was able to eat sufficiently at that time. Two years later, after self-interrupting treatment, he presented to our hospital with vomiting and jaundice. Due to the duodenal stenosis, endoscopic treatment was challenging, necessitating surgical intervention for the obstructed pancreatic and biliary stents. Triple bypass, including pancreaticojejunostomy, was performed instead of pancreaticoduodenectomy due to the patient's poor general condition and the development of collateral veins with superior mesenteric vein stenosis. Postoperatively, the patient experienced pancreatic fistula and fungal sepsis but was discharged from the hospital three months after surgery. Currently, his condition and nutritional status are good. Case 2 involves a 58-year-old man who was referred to our hospital due to pancreatic pseudocysts and pancreatic and biliary stenosis caused by alcoholic chronic pancreatitis. Endoscopic drainage for pancreatic pseudocysts and stenting for pancreatic and biliary stenosis were performed. After two years of follow-up, surgical treatment was performed due to the challenges posed by stenosis and multiple duodenal ulcers, which rendered endoscopic treatment difficult. Triple bypass, including longitudinal pancreaticojejunostomy with coring-out of the pancreatic head (Frey's procedure), was performed because pancreaticoduodenectomy was difficult due to severe inflammation and adhesions in the pancreatic head. His postoperative course was uneventful, and he was discharged 13 days after surgery. Triple bypass could serve as a viable alternative to pancreaticoduodenectomy in cases with poor general condition or a high risk of perioperative complications due to severe inflammation and adhesions.

## Introduction

The therapeutic goals of chronic pancreatitis (CP) are pain relief and the prevention/treatment of pancreatitis-related complications. Surgical intervention is indicated for patients with suspected malignancy or who are resistant to medical and endoscopic treatment. Surgical interventions for CP are generally classified into two procedures: pancreatic duct drainage and pancreatectomy [1]. Pancreatic duct drainage includes longitudinal (or lateral) pancreaticojejunostomy (LPJ), also called Partington's procedure [2], while pancreatic resection includes pancreaticoduodenectomy (PD), distal pancreatectomy, and duodenum-preserving resection of the head of the pancreas (Beger's procedure) [3]. Frey's procedure, a hybrid procedure combining LPJ with coring-out of the pancreatic head, integrates the advantages of both drainage and resection [4]. The procedure is considered to have better short- and long-term outcomes than PD [5,6] and is sometimes combined with other procedures such as DP [7].

In CP, irreversible progressive inflammation and fibrosis of the pancreas frequently cause stenosis or obstruction of the bile duct and the duodenum. PD is indicated for CP patients with biliary and duodenal stenosis [1,8]. However, it is occasionally difficult to perform PD due to a poor general condition or severe inflammation and adhesions caused by CP. Herein, we report two cases of CP complicated by biliary and duodenal stenosis who underwent triple bypass, a combination of biliary and gastric bypass and pancreatic duct drainage, with good outcomes.

## Case presentation

Case 1

A 51-year-old man presented to our hospital with vomiting and jaundice. He had a history of recurrent acute alcoholic pancreatitis progressing to CP, leading to biliary obstruction and pancreatic duct stenosis in the pancreatic head, for which he underwent endoscopic biliary and pancreatic stenting. However, he self-interrupted this treatment following a relocation two years prior to presenting to our hospital. Laboratory findings showed elevated hepatobiliary pancreatic enzymes and bilirubin, suggesting obstruction of the biliary and pancreatic stent (Table 1). There was no elevation in the tumor markers CEA (carcinoembryonic antigen) and CA 19-9 (cancer antigen 19-9).

**Table 1:** Patient's laboratory results in Case 1 WBC: white blood cells; RBC: red blood cells; HGB: hemoglobin; HCT: hematocrit; AST: aspartate aminotransferase; ALT: alanine aminotransferase; γGTP: gamma-glutamyl transferase; BUN: blood urea nitrogen; CRP: C-reactive protein.

Variable (Unit)	Result	Reference Range
WBC (×1000/μL)	11.4	3.3-8.6
RBC (×1000000/μL)	3.57	4.35-5.55
HGB (g/dL)	12	13.7-16.8
HCT (%)	35.3	40.7-50.1
Platelets (×1000/μL)	491	158-348
Total protein (g/dL)	8.3	6.6-8.1
Albumin (g/dL)	3.1	4.1-5.1
AST (U/L)	67	13-30
ALT (U/L)	76	10-42
Alkaline phosphatase	3584	115-359
γGTP (U/L)	242	13-64
Total bilirubin (mg/dL)	8	0.4-1.5
BUN (mg/dL)	26	8-20
Creatinine (mg/dL)	0.74	0.65-1.07
Sodium (mmol/L)	140	138-145
Potassium (mmol/L)	4	3.6-4.8
Chloride (mmol/L)	91	101-108
Amylase (U/L)	292	44-132
Lipase (U/L)	319	6-48
CRP (mg/dL)	7	0.00-0.14

**Figure 1:**
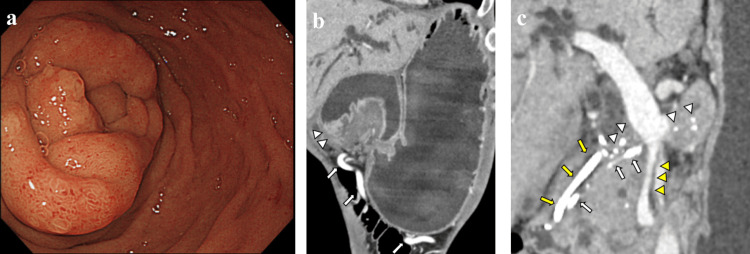
Findings of upper gastrointestinal endoscopy and preoperative CT imaging in Case 1 a: Upper gastrointestinal endoscopy showed an edematous and thick wall of the descending part of the duodenum. b: Preoperative CT imaging showed marked gastric dilatation, caliber changes in the duodenal bulb (white arrowheads), and developed collateral veins (white arrows). c: Diffuse pancreatic stones (white arrowheads) were observed, and a pancreatic stent (white arrows) and a biliary stent (yellow arrows) were placed for pancreatic and biliary stenosis. The superior mesenteric vein (SMV) on the dorsal side of the pancreas was narrowed (yellow arrowheads).

Upper gastrointestinal endoscopy showed edematous changes and thickening of the descending part of the duodenum, and the endoscope could not pass through the stenosis (Figure 1a). Gastric decompression with a nasogastric tube was necessary for duodenal stenosis. There was no structural atypia in the mucosa of the stenotic lesion, and the biopsy result was negative for malignancy. Computed tomography (CT) imaging showed marked gastric dilatation and caliber changes in the duodenal bulb (Figure 1b). Diffuse calcification throughout the pancreas, as well as swelling of the pancreatic head, mild dilatation of the main pancreatic duct, and multiple pseudocysts (the largest being 16 mm in diameter) with peripheral enhancement were observed around the uncinate process and the body of the pancreas. The common bile duct was dilated, the superior mesenteric vein (SMV) on the dorsal side of the pancreas was narrowed, and collateral veins had developed (Figures 1b, 1c).

Since endoscopic treatment was difficult due to the duodenal stenosis, surgical treatment was intended. His preoperative body mass index was 16.8 kg/m², and his serum albumin level was 1.9 g/dL, suggesting poor nutritional status. In addition, he had a fever, probably due to cholangitis. His laboratory data showed a white blood cell count of 23,300/µL with 93% neutrophils and total bilirubin of 8.4 mg/dL, which was persistently high. *Peptostreptococcus micros*, a gram-positive anaerobic coccus that usually exists in the gingival crevices and gastrointestinal tract, was detected in the blood culture. We administered ampicillin/sulbactam, and his fever subsided before the surgery. Due to the patient's poor general condition and the predictable surgical difficulty of severe organ adhesions, we planned to perform a triple bypass, including pancreatic drainage, instead of PD.

**Figure 2:**
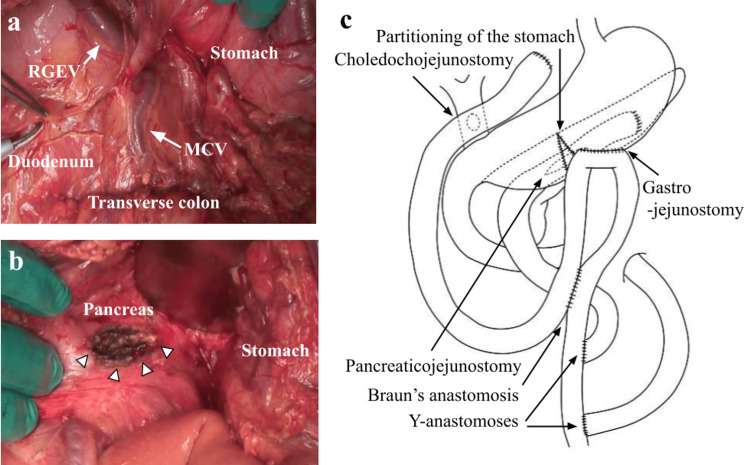
Operative findings in Case 1 a: The middle colic vein (MCV), the right gastroepiploic vein (RGEV), and other collateral veins were markedly distended due to the SMV stenosis. The outline of the pancreas was indistinct. b: A longitudinal incision along the main pancreatic duct was performed (white arrowheads). c: A triple bypass composed of choledochojejunostomy, gastrojejunostomy, and pancreaticojejunostomy was performed. Image Credits: Aya Noguchi

In the operation, a large amount of serous ascites and edematous changes in the intestinal tract and the greater omentum were observed. The pancreatic head, with marked inflammation, was lumped with the inferior vena cava, and mobilization of the duodenum was not possible because of the difficulty in dissecting them. In addition, the collateral veins were markedly distended due to SMV stenosis (Figure 2a). We made a longitudinal incision along the main pancreatic duct (Figure 2b) and transected the jejunum 30 cm anally from the ligament of Treitz. The distal stump of the disconnected jejunum was lifted retrocolically through the transverse mesocolon, and pancreaticojejunostomy was performed. The resection of the common bile duct was difficult due to the developed collateral veins. After the cholecystectomy, we transected the jejunum 30 cm anally from the pancreaticojejunostomy site and lifted the distal stump of the disconnected jejunum antecolically. Choledochojejunostomy with a side-to-side anastomosis was performed. For the gastrojejunostomy, the stomach was first partitioned from the greater curvature using an autosuture device. We performed antecolic gastrojejunostomy at the jejunum 40 cm anally from the choledochojejunostomy site and added jejunojejunostomy with a side-to-side anastomosis (Braun's anastomosis). A jejunojejunostomy with an end-to-side anastomosis (Y-anastomosis) was performed twice for the lifted jejunum of pancreaticojejunostomy and choledochojejunostomy (Figure 2c). The operation time was 514 minutes, and the amount of blood loss was 1760 g. Cytological examination of the intraoperatively obtained pancreatic fluid showed no malignant cells.

Following a postoperative pancreatic fistula and fungal sepsis caused by *Candida tropicalis*, he developed acute respiratory distress syndrome and disseminated intravascular coagulation but recovered with antifungal drugs and intensive care. Two months after surgery, he was transferred to a hospital for rehabilitation and was discharged three weeks after the transfer. For seven years since the surgery, no symptoms associated with CP have been observed, and there has been no evidence of pancreatic cancer. Nutritional status has improved, as evidenced by a 12 kg weight gain and a 1.7 g/dL rise in albumin levels since the preoperative period. Postoperative follow-up CT imaging showed improvement of the SMV stenosis.

Case 2

**Figure 3:**
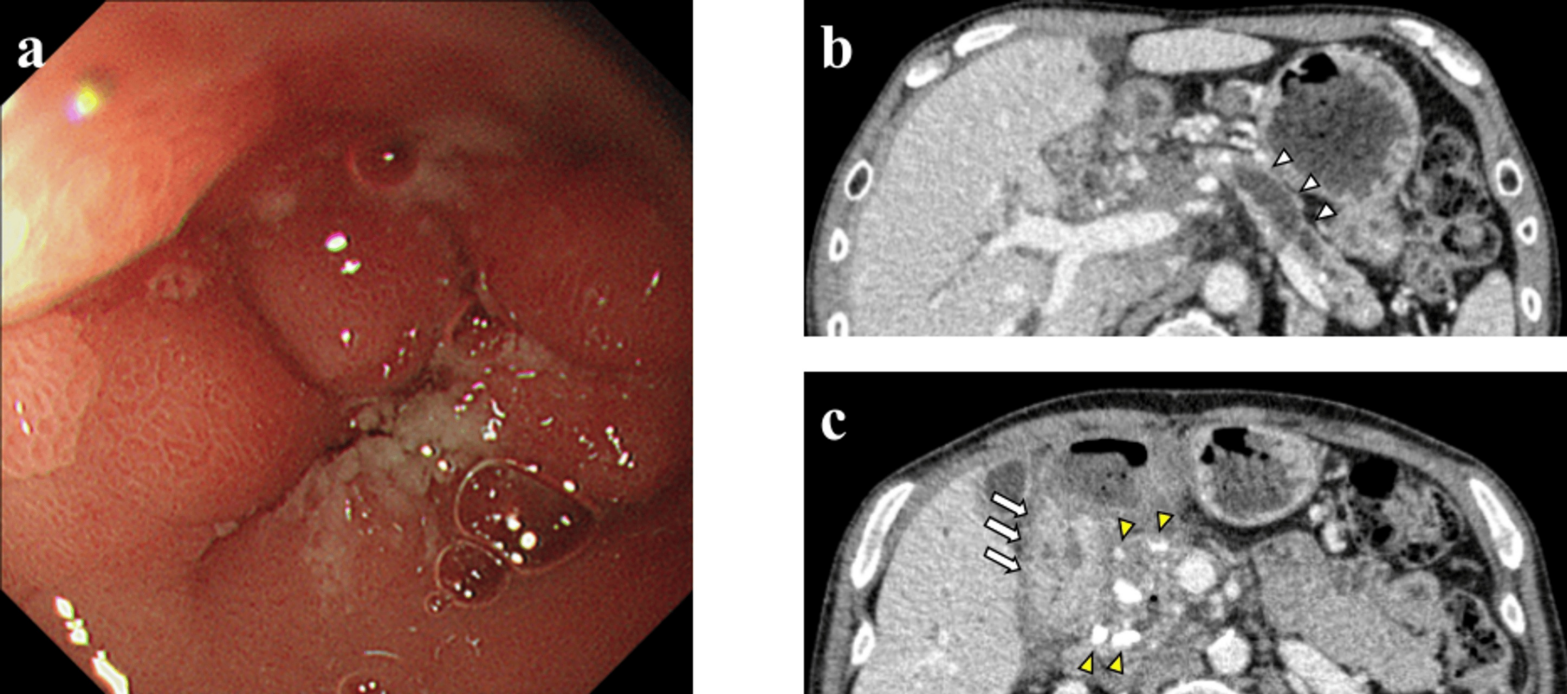
Findings of upper gastrointestinal endoscopy and preoperative CT imaging in Case 2 a: Upper gastrointestinal endoscopy showed duodenal stenosis due to ulcer scars. b: Preoperative CT imaging showed dilatation of the main pancreatic duct (white arrowheads). c: Pancreatic stones (yellow arrowheads) and thickening of the duodenal wall were observed (white arrows).

A 58-year-old man was referred to our hospital for treatment of a large pseudocyst in the pancreatic head and biliary stenosis. He developed alcoholic CP 12 years before and was started on insulin therapy for pancreatic diabetes mellitus two years prior. The internal drainage for the pancreatic pseudocyst and obstructive pancreatic/bile duct was performed using a transduodenal endoscopic puncture technique and internal stenting, respectively. Despite follow-up with repeated replacements of pancreatic or biliary stents, the endoscope procedures were complicated by duodenal stenosis caused by multiple ulcer scars in the duodenal bulb (Figure 3a). He was then referred to our department for surgery. The preoperative laboratory findings showed no elevation of hepatobiliary enzymes and tumor markers (CEA, CA19-9, DUPAN-2). He had no antibodies for *Helicobacter pylori* (*H. pylori*) and no history of regular use of nonsteroidal anti-inflammatory drugs (NSAIDs). CT imaging showed dilatation of the main pancreatic duct (Figure 3b), calcification in the pancreatic head and body, pseudocyst (up to 2 cm in diameter) in the pancreatic head, and thickening of the duodenum wall (Figure 3c). We planned to perform PD in case of detachable peripancreatic adhesions; otherwise, we would convert to a triple bypass.

**Figure 4:**
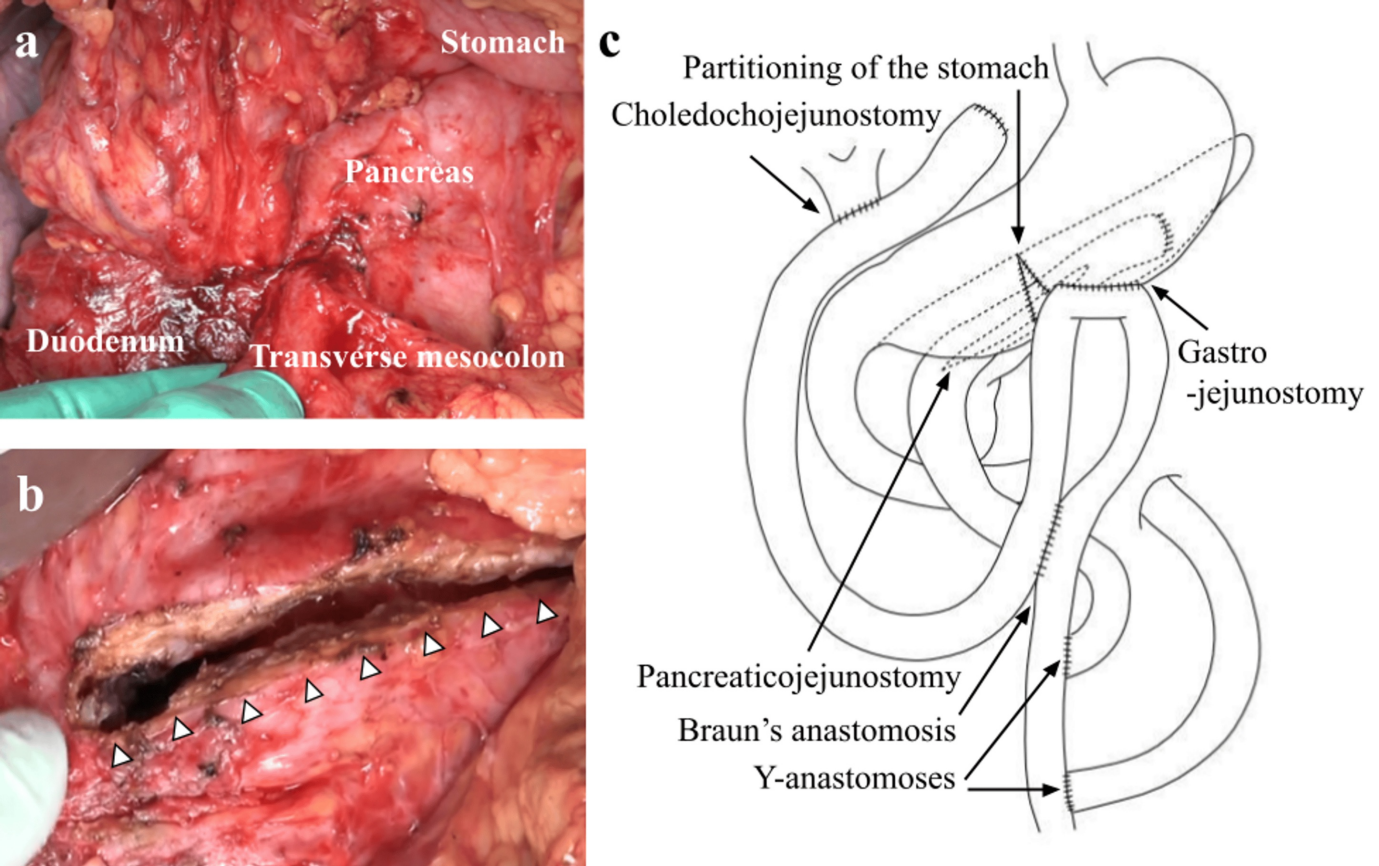
Operative findings in Case 2 a: The adhesions between the pancreas and the transverse mesocolon were hardened. b: A longitudinal incision was performed along the main pancreatic duct with coring-out of the pancreatic head (white arrowheads). c: Triple bypass composed of choledochojejunostomy, gastrojejunostomy, and longitudinal pancreaticojejunostomy was performed. Image Credits: Aya Noguchi

In the operative findings, the duodenum was not markedly deformed, but the duodenal bulb was hard due to ulcer scars. Although inflammation and adhesions around the pancreatic head were prominent, mobilization of the duodenum and the pancreatic head was possible. The transverse mesocolon was shortened due to adhesions caused by peripancreatic inflammation (Figure 4a). In addition, it was difficult to expose the SMV beneath the pancreas. Therefore, we decided to perform a triple bypass, including Frey's procedure. After cholecystectomy, we made a longitudinal incision along the main pancreatic duct and excised the pancreatic head (Figure 4b). Then, we transected the jejunum 30 cm anally from the ligament of Treitz and lifted the distal stump of the disconnected jejunum retrocolically through the transverse mesocolon; LPJ was performed. Subsequently, we transected the common bile duct and the jejunum 30 cm anally from the LPJ site. The distal stump of the disconnected jejunum was lifted retrocolically through the gap between the lifted jejunum for LPJ and the transverse mesocolon. The following procedures, such as end-to-side choledochojejunostomy, partitioning of the stomach, gastrojejunostomy, Braun's anastomosis, and two Y-anastomoses, were performed in the same manner as in Case 1. The operation time was 456 minutes, and the amount of blood loss was 513 g. Pathological examination of the cored-out pancreatic tissue revealed no malignancy. The patient had an uneventful postoperative course and was discharged 13 days after surgery. He is under outpatient follow-up and has had no symptoms, such as abdominal pain, for three years since surgery. There has been no evidence of pancreatic cancer.

## Discussion

We performed a triple bypass composed of choledochojejunostomy, gastrojejunostomy, and pancreatic duct drainage in two CP patients with biliary and duodenal stenosis, in whom PD was difficult to perform. For cases with a poor general condition or predictable difficulty in surgery due to severe inflammation and adhesions, triple bypass could be considered as an alternative to PD.

CP patients in Japan are predominantly male, and more than 70% of them are alcohol-related [9]. In CP, the progression of inflammation and fibrosis of the pancreas may result in stenosis of the surrounding tissues, such as the pancreatic duct, bile duct, duodenum, and portal vein system. Biliary and duodenal stenosis are found in 6% and 1.2% of patients hospitalized for CP, respectively, and some patients require surgical treatment [10]. Since duodenal ulcers are found in 14% of patients with alcoholic CP [11], an association between these two diseases has been suggested. In Case 2, CP may have affected the development of the duodenal ulcer because the two major causes of that, *H. pylori* infection and regular use of NSAIDs [12,13], were not recognized.

PD is generally performed for CP with biliary and duodenal stenosis [1,8]. PD has the advantage of local control of the lesion. Since CP patients have been reported to have a higher risk of developing pancreatic cancer [14], pancreatic resection should be performed when malignancy is suspected. However, PD can be excessively invasive for patients in poor general condition. Furthermore, there are also some cases where PD is difficult due to inflammation, adhesions, or portal vein stenosis. For patients like Case 1, where even the digestive tract reconstruction resulted in postoperative fungal sepsis, PD might be overly invasive and possibly fatal. Triple bypass, including pancreatic duct drainage, should be considered as an alternative procedure in cases such as ours.

We searched on PubMed and found three studies in which a similar surgery was performed from 1972 to 2024 with the search words "CP", "common bile duct", and "duodenal stenosis or duodenal obstruction" [15-17]. The findings from the aforementioned studies, along with our own, are presented in Table 2. Unlike the previous reports, we lifted the jejunum for LPJ and choledochojejunostomy, respectively, and performed gastrojejunostomy anally from choledochojejunostomy with the jejunum used for choledochojejunostomy. In our method, only gastrojejunostomy is passed before reaching choledochojejunostomy, which is considered a reconstruction method that can be easily treated endoscopically in cases of anastomotic stricture after choledochojejunostomy or choledocholithiasis.

**Table 2:** Reported cases of triple drainage procedure for simultaneous pancreatic, biliary, and duodenal stenosis due to chronic pancreatitis * Two jejunal limbs were elevated, one for pancreaticojejunostomy and the other for choledochojejunostomy and gastrojejunostomy. ND: not detected.

Case	Study	Age	Sex	Cause of chronic pancreatitis	Clinical presentation before surgery	Type of surgery	Pancreaticoenterostomy	Choledochoenterostomy	Gastroenterostomy	Pain-free term since surgery
1	Prinz et al. [15]	ND	ND	ND	ND	ND	Pancreaticojejunostomy at Roux-Y limb	Choledochoduodenostomy	Gastrojejunostomy at proximal jejunum	ND
2	Prinz et al. [15]	ND	ND	ND	ND	ND	Pancreaticojejunostomy at Roux-Y limb	Choledochoduodenostomy	Gastrojejunostomy at proximal jejunum	ND
3	Prinz et al. [15]	ND	ND	ND	ND	ND	Pancreaticojejunostomy at Roux-Y limb	Choledochojejunostomy at Roux-Y limb	Gastrojejunostomy with subtotal gastrectomy	ND
4	Sugerman et al. [16]	ND	ND	ND	ND	ND	Pancreaticojejunostomy at Roux-Y limb	Choledochojejunostomy at Roux-Y limb	Gastrojejunostomy at Roux-Y limb	3 years
5	Abe et al. [17]	55	Male	Alcohol	Poor oral intake	ND	Pancreaticojejunostomy at Roux-Y limb	Choledochojejunostomy at Roux-Y limb	Gastrojejunostomy at proximal jejunum with Braun's anastomosis between choledochojejunostomy and pancreaticojejunostomy	5 years
6	Present Case 1	51	Male	Alcohol	Vomiting, jaundice, fever	Emergency	Pancreaticojejunostomy at Roux-Y limb*	Choledochojejunostomy at Roux-Y limb	Gastrojejunostomy with partitioning at Roux-Y limb with Braun's anastomosis between choledochojejunostomy and gastrojejunostomy	5 years
7	Present Case 2	58	Male	Alcohol	None	Elective	Pancreaticojejunostomy at Roux-Y limb*	Choledochojejunostomy at Roux-Y limb	Gastrojejunostomy with partitioning at Roux-Y limb with Braun's anastomosis between the choledochojejunostomy and gastrojejunostomy	6 months

Abe et al. [17] performed Braun's anastomosis between choledochojejunostomy and LPJ. We added Braun's anastomosis between choledochojejunostomy and gastrojejunostomy because it may reduce the risk of gastric cancer due to long-term exposure to bile juice. Moreover, partitioning of the stomach was performed in the hope that orally ingested food would flow more easily through the gastrojejunostomy. The postoperative courses in the reported cases were uneventful, and triple bypass is considered a safe and useful treatment in cases where PD is difficult to perform. Because Frey's procedure was reported to be equally effective in pain control and superior in terms of postoperative complications and preservation of pancreatic function to PD [18-20], triple bypass, which includes coring-out of the pancreatic head, might possibly be more effective than PD for CP with biliary and duodenal stenosis. It should be noted that since there is a risk of developing pancreatic cancer in the remaining pancreas, postoperative follow-up is important.

To demonstrate the efficacy of triple bypass, further accumulation of cases and evaluation of postoperative pain and pancreatic function are essential. However, we consider that this surgical technique could be effective, at least in cases in which PD is difficult.

## Conclusions

In CP patients with biliary and duodenal stenosis, triple bypass appears to offer advantages over PD in terms of reduced surgical invasiveness, potentially leading to increased safety, and preservation of pancreatic function due to sparing of pancreatic tissue. We believe that triple bypass may be a more suitable option than PD in certain cases, particularly in those with severe inflammation and adhesions.
